# Elaboration and Validation of the Medication Prescription Safety Checklist^1^

**DOI:** 10.1590/1518-8345.1817.2921

**Published:** 2017-08-03

**Authors:** Aline de Oliveira Meireles Pires, Maria Beatriz Guimarães Ferreira, Kleiton Gonçalves do Nascimento, Márcia Marques dos Santos Felix, Patrícia da Silva Pires, Maria Helena Barbosa

**Affiliations:** 2 MSc.; 3 Post-doctoral fellow, Universidade Federal do Triângulo Mineiro, Uberaba, MG, Brazil.; 4 MSc, RN, Hospital de Clínicas, Universidade Federal do Triângulo Mineiro, Uberaba, MG, Brazil.; 5 Doctoral student, Universidade Federal do Triângulo Mineiro, Uberaba, MG, Brazil. Scholarship holder at Coordenação de Aperfeiçoamento de Pessoal de Nível Superior (CAPES), Brazil.; 6 PhD, Adjunct Professor, Instituto Multidisciplinar em Saúde, Universidade Federal da Bahia, Vitória da Conquista, BA, Brazil.; 7 PhD, Associate Professor, Universidade Federal do Triângulo Mineiro, Uberaba, MG, Brazil.

**Keywords:** Patient Safety, Medication Errors, Drug Prescriptions

## Abstract

**Objective:**

to elaborate and validate a checklist to identify compliance with the recommendations for the structure of medication prescriptions, based on the Protocol of the Ministry of Health and the Brazilian Health Surveillance Agency.

**Method:**

methodological research, conducted through the validation and reliability analysis process, using a sample of 27 electronic prescriptions.

**Results:**

the analyses confirmed the content validity and reliability of the tool. The content validity, obtained by expert assessment, was considered satisfactory as it covered items that represent the compliance with the recommendations regarding the structure of the medication prescriptions. The reliability, assessed through interrater agreement, was excellent (ICC=1.00) and showed perfect agreement (K=1.00).

**Conclusion:**

the Medication Prescription Safety Checklist showed to be a valid and reliable tool for the group studied. We hope that this study can contribute to the prevention of adverse events, as well as to the improvement of care quality and safety in medication use.

## Introduction

In recent years, concerns with patient safety have emphasized the aspect of risk management associated with medication use^1^. Medicines are products capable of preventing, diagnosing, curing illnesses or relieving symptoms, but countless errors occur in the medication treatment process the patients receive^2^. 

One of the main adverse events hospitalized patients are victims of are medication errors, representing a severe problem in health services, besides being frequent^3^ ^-^ ^4^ and common in all health institutions due to the complexity of the process. It can happen in the prescription, dispensing or administration of medicines and is established as one of the causes of iatrogenic effects^5^.

The *Conselho Nacional de Coordenação de Relatórios e Prevenção de Erros de Medicamentos* (National Coordinating Council for Medication Error Reporting and Prevention - NCCMERP) defines medication error as an avoidable event, which can lead to the bad use of medication or to patient damage while the patient is under the professional’s control^6^.

Based on an analysis of the contribution of medical errors to deaths in the United States of America, it was estimated through research that medical errors can represent approximately 251 thousand deaths per year in the country, ranking third. Error is considered as an unintentional act, which did not produce the desired outcome, as well as execution or planning errors or failures in the care process^7^. 

The most sensitive method to identify medication dispensing and administration errors is observation, while the review of records is considered more appropriate to identify errors in medication prescriptions^8^. Among the different medication errors, prescription errors stand out due to their potential to cause harmful consequences to the patients^9^ and because they represent a considerable proportion of avoidable drug-related problems^10^.

The prescription process is complex and permeated by errors^11^. Prescription errors happened in 14.7% of the medication prescriptions in the United Kingdom, the most common being omission, wrong dose and incomplete prescription^12^.

The medical prescription is the reference document that guides and influences the other phases of the medication process. It is an essential communication tool among health professionals^11^ and plays an important role in the prevention and occurrence of errors^13^.

An analysis of systematic reviews to determine the effects of hospital technologies on the quality, safety and efficacy of care demonstrated that, for the electronic prescriptions, substantially lesser evidence of medication errors was found, as well as greater compliance with the guidelines and better control of illnesses and better response time to the dispensing^14^. 

The prescriptions should be comprehensive, in terms of the existence of information needed for all professionals who use them, as omitting information from the prescription can contribute to the occurrence of errors^13^. It should be kept in mind that error reporting by all health professionals, in combination with organizational changes, can favor patient safety and minimize medical errors^7^. 

The engagement of different professionals in the various phases of the medication prescription process is essential, as reports on the occurrence of possible errors represent a possibility for learning, for the implementation of preventive measures, for high-quality care provision and for patient safety promotion through medication governance^15^.

To reduce the incidence of adverse events in public and private health services and promote safe medication usage practices, in the Brazilian literature, the Medication Prescription, Usage and Administration Safety Protocol stands out, which addresses safe practices for medication prescription, distribution and administration^16^. 

In view of the need for studies that identify the absence of information from the prescriptions and the lack of instruments in the literature, in this study, we intended to answer the following question: does a checklist permit verifying compliance with the safety recommendations concerning the structure of medication prescriptions?

Hence, considering that medication errors compromise the quality of care and patient safety, in this study, we aimed to elaborate a checklist to identify the compliance with recommendations for the structure of medication prescriptions, as well as to carry out the face and content validation and the reliability analysis.

## Method

A methodological research was developed in three phases: construction of a tool to verify the safety in the medication prescription, face and content validation and reliability analysis. 

To construct the instrument, the recommendations of the Medication Prescription, Usage and Administration Safety Protocol were used, coordinated by the Brazilian Health Department and the Brazilian Health Surveillance Agency (Anvisa), in partnership with *Fundação Oswaldo Cruz* (Fiocruz) and *Fundação Hospitalar do Estado de Minas Gerais* (Fhemig)^16^. The tool constructed was called *Lista de Verificação de Segurança na Prescrição de Medicamentos* (Medication Prescription Safety Checklist - LVSPM) and covers identification data of the prescription and its medicines.

For the face and content validation, five multiprofesional judges were selected, being: one physician, one pharmacist and three nurses, all of whom held a Ph.D. and were experienced in the theme area of the research, four of them being faculty members at federal universities.

Initially, the judges were contacted by e-mail, inviting them to participate in the content validation phase of the LVSPM. After they had agreed, a document was forwarded with the description, purpose and objectives of the research, as well as the instrument, in order to assess whether it is measuring what it is intended to measure (face validation) and the relevance of each item in the construct studied (content validation)^17^, that is, if both properly represent the hypothetic universe of the object, i.e. patient safety in medication prescription.

The reliability analysis was verified by means of the interrater method, by comparing two nurses’ independent observations in the use of the checklist. The observations were made after training on the instrument and its applicability.

The study was developed at the medical and surgical clinical wards of a public teaching hospital in Uberaba, a city located in the interior of Minas Gerais, with a capacity of 37 and 65 beds, respectively. The choice of the wards was based on the feasibility criterion of the research, as they presented a computerized prescription system and a larger volume of prescriptions.

In the calculation of the sample size for the interrater reliability analysis, an expected Intraclass Correlation Coefficient (ICC) of 0.90 between the scores was considered, admitting coefficients not lower than ICC=0.75 for a 90% power, significance being set at α=0.05. Using the application PASS 2002 (Power Analysis and Sample Size), with these *a priori* coefficients, a sample size of n=36 prescriptions was obtained. Considering a 10% sampling loss, the maximum number of attempts would be 40. Nevertheless, considering the losses in the data collection period, the final number of prescriptions analyzed was 27.

A pilot study was developed, using 15 prescriptions, to verify the adequacy of the validated checklist’s contents to the reality of the information collection at the institution. Nevertheless, the measuring properties of the tool were not subject to statistical analysis in this phase, as the form and structure of the collection instrument underwent no changes.

The data were collected between July and September 2015, after the Medical Filing Service (Same) had provided the printed prescriptions. The LVSPM was applied to the medication prescriptions of the units studied in order to identify compliance with the recommendations of the Health Department and Anvisa’s Protocol. It is highlighted that, before the start of the data collection process, the judges were submitted to training for the sake of conformity of the data collection.

In the data analysis, the categorical variables were subject to univariate analyses through absolute and relative frequency tables. The interrater reliability was verified through the Kappa coefficient in the first part of the tool, as the variables are dichotomous, considering the correlation based on the magnitude of the agreement as low (0-0.20), regular (0.21-0.40), moderate (0.41-0.60), substantial (0.61-0.80) and almost perfect (≥0.81)^18^, and the intraclass correlation coefficient as adequate when >0.70^19^ in the second part, in view of the quantitative nature of the variable. Significance was set at 0.05.

Approval for the research project was obtained from the Research Ethics Committee (Protocol 1.012.450), in compliance with Resolution 466/2012, which waived the signing of the Free and Informed Consent Form.

## Results

The elaboration of the checklist was based on the items proposed in the protocol concerning the prescription, resulting in a first version with 27 items, divided in two parts: identification of the prescription (11 item) and medicines in the prescription (16 items).

**Figure 1:**
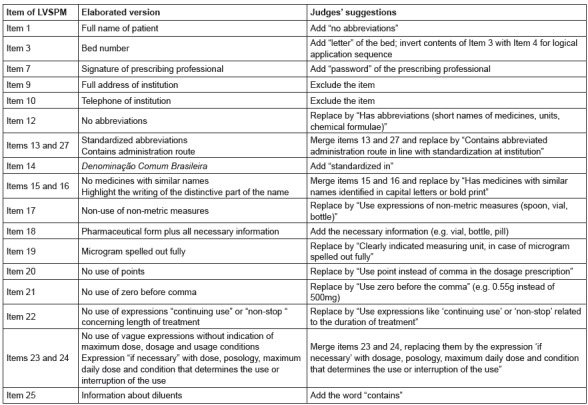
Changes suggested by expert committee to create the final version of the Medication Prescription Safety Checklist. Uberaba, MG, Brazil, 2015

The face and content validation of the LVSPM were verified by means of the interrater agreement, with a minimum consensus of 80%. The judges analyzed this first version of the tool and their suggested modifications are displayed in Figure 1.

**Figure 2:**
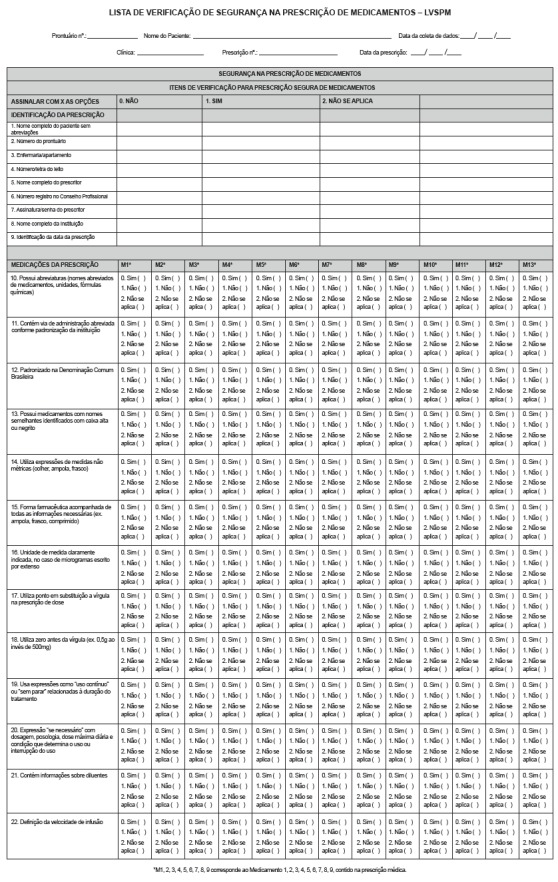
Final version of the Medication Prescription Safety Checklist. Uberaba, MG, Brazil, 2015

All of the experts’ suggestions were executed because they were pertinent. After the changes, the final version of the LVSPM (Figure 2) contained 22 items, as items 9 and 10 were excluded and items 16, 24 and 27 were merged. 

The first part of the checklist refers to the identification items of the medication prescription and contains nine items with three alternative answers and their respective codes: no (0); yes (1); does not apply (2), marking the most appropriate alternative with an X.

The second part, in turn, consists of 13 items related to the prescribed drugs, so that the code M represents the medicine and an Arabic numeral its order in the prescription, e.g. M1 corresponds to Medicine 1 and so forth, according to the number (n) of medicines in the prescription. The alternative answers are codes 0, 1, and 2, which mean non-compliance, compliance and does not apply, respectively. The alternatives yes and no can receive code 1, depending on the items that are to be assessed. 

To determine the compliance score, the answers with score 1 (one) are added up, according to the following formula: general compliance = sum of total compliance percentages/total number of valid items. It is highlighted that, for items 1 to 9, codes 1 are converted to 100%. For items 10 to 22, the compliance proportion is calculated by the sum of code 1, divided by the number of valid items (total number of medicines - blank items), multiplying this result by 100. The checklist score ranges between 0 and 100, with higher scores indicating greater compliance.

It should be clarified that items 10, 14, 17, 18 and 19 are considered inverted items. That is due to the fact that, the higher the instrument score, the greater the compliance with the recommendations. To calculate the compliance score, these items were scored 0 when the answer was yes and 1 when it was no. Thus, both options (yes and no) can receive code 1, depending on the items that are to be assessed.

The reliability analysis was evidenced by means of the Kappa and ICC coefficients. In the first part of the checklist, items Q1 to Q9 were analyzed according to the results described in Table 1.

**Table 1:** Results of the interrater reliability analysis for items Q1 to Q9: identification of the prescription. Uberaba, MG, Brazil, 2015

**Identification of prescription**	**Judge A**						**Judge B**					PC*	***K*** ^**†**^	***p***
	**No**			**Yes**			**No**			**Yes**				
	**N**	**%**		**N**	**%**		**N**	**%**		**N**	**%**	**%**		
**Q1. Full name of patient without abbreviations**	**0**	**0**		**27**	**100**		**1**	**3.7**		**26**	**96.3**	**100**	**1.00**	**<0.001**
**Q2. Patient file number**	**0**	**0**		**27**	**100**		**1**	**3.7**		**26**	**96.3**	**100**	**1.00**	**<0.001**
**Q3. Nursing ward/apartment**	**0**	**0**		**27**	**100**		**0**	**0**		**27**	**100**	**100**	**-**	**-**
**Q4. Bed number/letter**	**0**	**0**		**27**	**100**		**0**	**0**		**27**	**100**	**100**	**-**	**-**
**Q5. Full name of prescribing professional**	**0**	**0**		**27**	**100**		**0**	**0**		**27**	**100**	**100**	**-**	**-**
**Q6. Professional board registration number**	**0**	**0**		**27**	**100**		**0**	**0**		**27**	**100**	**100**	**-**	**-**
**Q7. Signature/password of prescriber**	**0**	**0**		**27**	**100**		**1**	**3.7**		**26**	**96.3**	**100**	**1.00**	**<0.001**
**Q8. Full name of institution**	**27**	**100**		**0**	**0**		**27**	**100**		**0**	**0**	**100**	**-**	**-**
**Q9. Identification of prescription date**	**0**	**0**		**27**	**100**		**0**	**0**		**27**	**100**	**100**	**-**	**-**

*Agreement proportion.

†Kappa coefficient.

According to Table 1, the agreement index was perfect for items Q1, Q2 and Q7 (K=1.00), with a statistically significant difference (p<0.001). 

Among the nine first items assessed, the Kappa coefficients and significance levels were not calculated for six (Q3, Q4, Q5, Q6, Q8 and Q9), as the results of the interrater agreement did not constitute a squared matrix. 

The agreement proportion corresponded to 100% for all items in the first part of the instrument, that is, the judges agreed on all items of the 27 prescriptions analyzed.

Table 2 evidences the reliability analysis of the second part of the instrument, medicines on the prescription (items Q10 till Q22).

**Table 2:** Results of interrater reliability analysis for items Q10 till Q22: medicines on the prescription. Uberaba, MG, Brazil, 2015

Medicines on the prescription	Judge A			Judge B		ICC*	p
	Mean	sd		Mean	sd		
Q10. Has abbreviations^†^	29.65	21.92		29.65	21.92	1.00	-
Q11. Contains abbreviated administration route as standardized	100.00	0.00		100.00	0.00	-	-
Q12. Standardized in *Denominação Comum Brasileira*	100.00	0.00		100.00	0.00	-	-
Q13. Has medicines with similar names	-	-		-	-	-	-
Q14. Uses non-metric measures^†^	56.09	31.62		54.98	30.78	0.99	<0.001
Q15. Pharmaceutical form accompanied by all necessary information	0.00	0.00		0.00	0.00	-	-
Q16. Clearly indicated measuring unit	55.68	32.30		54.57	31.00	0.99	<0.001
Q17. Uses point to replace comma in dose prescription^†^	100.00	0.00		100.00	0.00	-	-
Q18. Uses zero before the comma^†^	100.00	0.00		100.00	0.00	-	-
Q19. Uses expressions like “continuing use” or “non-stop”^†^	91.41	11.76		89.81	12.40	0.85	<0.001
Q20. Expression “if necessary” with all necessary information	0.00	0.00		0.00	0.00	-	-
Q21. Contains information about diluents	46.53	32.54		47.63	33.24	0.92	<0.001
Q22. Definition of infusion speed	38.09	40.53		38.09	40.53	1.00	-

*Intraclass Correlation Coefficient.

†Yes=0 / No=1 (to calculate compliance score).

The data evidence that the ICC and significance level of items Q11, Q12, Q15, Q17, Q18 and Q20 could not be calculated as there was no variation among the observers, despite complete agreement.

Items Q14 (ICC=0.99), Q16 (ICC=0.99), Q19 (ICC=0.85), Q21 (ICC=0.92) presented adequate reliability (ICC>0.85) and are statistically significant (p<0.001). Items Q10 and Q22 presented ICC=1.00, with excellent reliability. Item Q13 (has medicines with similar names) did not present a compliance score as, in this item, for all medicines, code 2 (does not apply) was marked. Nevertheless, this item was not excluded because none of the prescriptions analyzed contained medicines with similar names, but these can be found at another time. As observed, the reliability of the LVSPM was excellent and statistically significant (p<0001).

## Discussion

Other research results evidence the importance of using tools that permit the identification of possible prescription errors, contributing to improve the medication administration process, which involves different health professionals of relevant importance for the nursing team.

In one study, it was affirmed that prescribing correctly represents one of the essential skills to guarantee patient safety and, therefore, 74 medicine students were assessed in a study of the number of prescription errors committed in a prescription test. These tests were assessed by means of a checklist to identify the prescription errors, evidencing that the students committed 69% of errors^10^. 

In another study, the impact of introducing a prescription verification and correction checklist on the quality and safety of hospital prescriptions was assessed at two pediatric nursing wards of a university hospital in London, England. The technical and clinical prescription errors were assessed before and after the introduction of the checklist. The global technical error rate in the pre-intervention period was 10.8% and the clinical error rate 4.7%. The most common errors were: lack of contact details for the physician and dose omissions. After the implementation of the verification and correction checklist, the error rates corresponded to 7.3 and 5.5%, respectively. As for the clinical error, no significant impact of the intervention was detected. The researchers concluded that the implementation of a verification and correction checklist led to improvements in the quality of written prescriptions^20^.

A Chilean research also aimed to adapt and validate two checklists, one to measure the errors in handwritten prescriptions and the other to detect errors in the medication preparation process. The instruments were submitted to three phases: adaptation, as the instruments were based on the error classification of the National Coordinating Council for Medication Error Reporting and Prevention (NCCMERP); review by experts and reliability analysis. The checklists for medication prescription and dispensing consisted of 12 items to measure the prescription errors and seven to measure the preparation errors. The instruments showed to be valid and reliable^21^. 

To reduce the prescription errors, a French study is also highlighted, a pioneer in the development of a preliminary screening tool to identify omissions and inappropriate prescriptions in pediatrics, based on international and French guidelines^5^. 

In a different study, aiming to explore factors that provoke prevalent errors in hospitals tending towards medication errors, raising awareness about their existence and offering recommendations on how to minimize them to improve patient safety, 162 valid histories were analyzed for patients hospitalized at a public hospital in Ghana, based on a checklist to register possible medication errors. The results evidenced that: 60.5% of the patients did not receive the actual quantity of medicines; illegible writing; similar medication packages and labels; crowded workspace, besides distractions such as telephone ringing, interruption of one task to perform another and unnecessary conversation among the staff. The study highlighted the vulnerability of the medication process at the hospital in terms of medication errors and emphasized that, as part of a medication safety process, the hospitals should implement incident registration mechanisms, as a means to prevent the recurrence of medication errors^22^. 

In another study, the importance of reporting the medication errors was highlighted, as this represents a possibility to learn and implement preventive measures. In addition, the need for the safety culture in institutions was emphasized, where medication governance promotes patient safety and high-quality care provision^15^. 

The use of the LVSPM is recommended as a management tool in the nurses’ clinical practice, offering support for the implementation of evidence-based care. The recommendations of a literature review corroborate this assertion, identifying evidence-based health care as a subculture of the patient safety culture. The best evidence-based practices include standardized processes, protocols, checklists and orientations, aspects that favor the safety culture^23^. 

The application of a checklist like the LVSPM in daily work and the careful analysis of the results obtained can significantly improve the quality and safety of the medication therapy provided to the patients, besides guiding the professionals, especially the nurses to eliminate the errors deriving from the medication process^24^.

We consider that the predictive validity represents a limitation in this study. It should be highlighted though that this tool can be used in a subsequent study with a longitudinal and prospective design to estimate the predictive validity of complying with the recommendations, using the occurrence or not of medication-related adverse events as a criterion. We also consider that the LVSPM items refer to the practice at any healthcare level. Hence, future studies are needed to assess the use of the checklist in other contexts beyond the hospital. 

## Conclusion

In view of the above, the validity and reliability of the LVSPM were demonstrated. The checklist can be used in clinical practice, permitting the identification of prescription errors by nurses and other health professionals. 

Judgments on the applicability in clinical practice depend on further research in different contexts. The LVSPM is a management instrument for clinical practice that can further the understanding of the needs to improve the prescriptions, resulting in the better quality of care, patient safety, evidence-based decision making by nurses and the reduction of medication-related errors. 
